# Differential expression of centrosomal proteins at different stages of human glioma

**DOI:** 10.1186/1471-2407-10-268

**Published:** 2010-06-09

**Authors:** Joon-Khim Loh, Ann-Shung Lieu, Chia-Hua Chou, Fang-Yi Lin, Chia-Hung Wu, Sheng-Long Howng, Chung-Ching Chio, Yi-Ren Hong

**Affiliations:** 1 Department of Neurosurgery, Kaohsiung Medical University Hospital, Kaohsiung, Taiwan; 2 Faculty of Medicine, Graduate Institute of Medicine, College of Medicine, Kaohsiung Medical University Hospital, Kaohsiung, Taiwan; 3 Department of Neurosurgery, Chi Mei Medical Center, Tainan 710, Taiwan; 4 Faculty of Medical Information Management, College of Health Science, Kaohsiung Medical University, Kaohsiung, Taiwan; 5 Department of Biochemistry, Faculty of Medicine, College of Medicine, Kaohsiung Medical University, Kaohsiung, Taiwan

## Abstract

**Background:**

High-grade gliomas have poor prognosis, requiring aggressive treatment. The aim of this study is to explore mitotic and centrosomal dysregulation in gliomas, which may provide novel targets for treatment.

**Methods:**

A case-control study was performed using 34 resected gliomas, which were separated into low- and high-grade groups. Normal human brain tissue was used as a control. Using immunohistochemical analysis, immunofluorescent microscopy, and RT-PCR, detection of centrins 1 and 2, γ-tubulin, hNinein, Aurora A, and Aurora B, expression was performed. Analysis of the GBM8401 glioma cell line was also undertaken to complement the *in vivo *studies.

**Results:**

In high-grade gliomas, the cells had greater than two very brightly staining centrioles within large, atypical nuclei, and moderate-to-strong Aurora A staining. Comparing with normal human brain tissue, most of the mRNAs expression in gliomas for centrosomal structural proteins, including centrin 3, γ-tubulin, and hNinein isoforms 1, 2, 5 and 6, Aurora A and Aurora B were elevated. The significant different expression was observed between high- and low-grade glioma in both γ-tubulin and Aurora A mRNA s. In the high-grade glioma group, 78.6% of the samples had higher than normal expression of γ-tubulin mRNA, which was significantly higher than in the low-grade glioma group (18.2%, p < 0.05).

**Conclusions:**

Markers for mitotic dysregulation, such as supernumerary centrosomes and altered expression of centrosome-related mRNA and proteins were more frequently detected in higher grade gliomas. Therefore, these results are clinically useful for glioma staging as well as the development of novel treatments strategies.

## Background

Gliomas are common brain cancers that are notoriously hard to treat. High-grade gliomas are especially difficult, and their prognosis is poor. Standard treatment for high-grade gliomas is limited to resection followed by radio/chemotherapy, resulting in a median survival of 14 months [1]. Therefore, the development of novel, targeted therapies is the best hope for glioma patients.

In recent years, rapid advances in understanding the role of mitotic dysregulation as a key oncogenic event have been reported. A number of cell cycle checkpoints exist at the mitosis phase of the cell cycle to ensure that chromosome segregation occurs in a timely and orderly fashion and that the correct number of centrioles and chromosomes are segregated into the two daughter cells [2]. If mitosis becomes dysregulated in a cell often due to centrosome abnormalities, aneuploidy may result, which may contribute to cellular transformation [2]. Although it is unknown whether centrosome abnormalities induce cellular transformation or result as a consequence of it, detection of centrosome defects in early-stage cancers supports the notion that they may directly contribute to transformation [2].

Increased knowledge of mitotic regulation in normal and cancerous cells has resulted in the development of drugs against these new targets [3,4]. A number of mitotic regulatory proteins, including Checkpoint with forkhead and ring finger domains (CHFR), Aurora A (also known as serine/threonine kinase 15 [STK15]), Aurora B, Aurora C, Polo-like kinases (Plk1-4), and Nek kinases (NIMA1-11) [5,6] as well as structural proteins of the centrosome, such as γ-tubulin, centrin 2, centrin 3, pericentrin, and hNinein have been identified [2,7,8]. Although genetic and epigenetic changes that result in mitotic dysregulation have been identified in various cancer cells [2], few studies have assessed it in gliomas [9-14]. Recently, a large genome-wide association study (GWAS) of 1,878 glioma cases versus 3,670 controls was undertaken [15,16]. Five critical susceptibility loci for glioma were identified, one of which was 20q13.33 [17], which is very near the locus for STK15/Aurora A located at 20q13.2-q13.3 http://www.ncbi.nlm.nih.gov/gene/6790?ordinalpos=5&itool=EntrezSystem2.PEntrez.Gene.Gene_ResultsPanel.Gene_RVDocSum. Further analysis of 692 high-grade gliomas versus 3,992 controls in the GWAS identified the RTEL gene, which is involved in regulation of homologous recombination, as a putative gene at the 20q13.33 locus associated with high-grade gliomas rather than Aurora A [16]. Although these data serve to reinforce the importance of this region of the genome and the potential association of Aurora A with high-grade glioma, the inconsistent results from various groups are a reminder that this research is at the early stages. In other cancer types, data is accumulating that Aurora A is a good prognostic indicator [16-19].

Other centrosomal structural proteins, such as hNinein, centrin, and pericentrin, may influence spindle body assembly during mitosis and are overexpressed in malignant tumors [7,8,20]. For example, Pihan *et al*. [21] selectively induced centrosome abnormalities by elevating pericentrin levels in prostate epithelial cell lines, which replicated many phenotypic characteristics associated with tumor-like prostate carcinoma. Pericentrin and γ-tubulin assemble into a unique centrosome lattice, which acts as a higher order organization of microtubule nucleating sites at the centrosome [22].

To test the hypothesis that altered expression of centrosome-related proteins may contribute to glioma grade, we analyzed the expression levels of centrosome regulatory proteins, such as Aurora A, and the chromosomal passenger protein, Aurora B, as well as centrosome structural proteins, including centrins 1 and 2, γ-tubulin, and hNinein in 34 glioma samples. In addition, high- and low-grade gliomas were compared to identify specific alterations that may facilitate glioma staging as well as provide novel targets for treatment. Finally, the glioma cell line, GBM8401, was also analyzed for centrosome defects.

## Methods

### Tissue collection

Brain tumor samples were obtained from patients undergoing surgery or biopsy at either the Kaohsiung Medical University Hospital or the Chi-Mei Medical Center in Taiwan. The tumors were classified according to the 1993 WHO classification [23]. Normal human brain tissues were purchased from Clontech laboratories (Mountain View, CA); the tissues were isolated from 8 male Caucasians (ages 43-65). Glioma tissue samples were collected fresh at the time of surgery, snap frozen in liquid nitrogen, and stored frozen at -135°C. The presence of cancer tissue and histological grade was confirmed by a trained pathologist.

This study used only excess tissue from medically-necessary surgeries and excess diagnostic samples; it was approved by the Institutional Review Board of the Chung-Ho Memorial Hospital, Kaohsiung Medical University (KMUH-IRB-960237 and KMUH-IRB-960435). In addition, written informed consent was obtained from all patients. This study was done in accordance with the principles outlined in the Declaration of Helsinki.

### RNA isolation

RNA was isolated with TRIzol reagent (Gibco-BRL, Rockville, MD) following the manufacturer's instructions. Briefly, 100 mg of tissue was homogenized in 1 mL of TRIzol reagent and mixed with 0.2 mL of guanidinium phenol/chloroform. After centrifugation (12,000 × *g*, 15 min, 4°C), RNA was recovered from the aqueous layer by precipitation with isopropyl alcohol. After the RNA was washed in 75% ethanol, it was air-dried and resuspended in DEPC-treated water.

### RT-PCR analysis

Reverse transcriptase PCR was performed following standard protocols. To generate cDNA, 5 μg RNA was incubated in a reaction mixture containing 50 mM Tris-HCl (pH 8.3), 10 mM DTT, 10 mM KCl, 0.5 mM dNTPs, 30 μg RNAsin (Promega, Madison, WI), 0.8 units of Superscript II reverse transcriptase (Gibco-BRL), and water to 20 μL for 1 h at 37°C.

The PCR reactions consisted of 2 μL of cDNA in a 50 μL reaction containing 50 mM Tris-HCl (pH 9.2), 16 mM (NH_4_)_2_SO_4_, 1.75 mM MgCl_2_, 10% DMSO, 0.3 mM of each primer, and 5 units of Pwo DNA polymerase (Boehringer Mannheim, Mannheim, Germany). The PCR conditions included denaturation at 95°C for 1 min followed by 30 cycles of 95°C for 30 sec, 58°C for 60 sec, and 68°C for 25 sec and one final extension at 68°C for 10 min. The following PCR primer sets were used:

γ-tubulin: forward, 5' CTCAAGAGGCTGACGCAGAAT 3' and reverse, 5' CTGGCTGACATGATGGTAGACAC 3'; centrin 2: forward, 5' CGGGAAGCTTTTGATCTTTTCGATGCG 3' and reverse, 5' GCTGGTCTTTTTCATGATGCG 3'; centrin 3: forward, 5' TTAAATGTCACCAGTCATAATAGC 3' and reverse, 5' AATGAGTTTAGCTCTGAGAAGT 3'; Aurora A: forward 5' GCTGGAGAGCTTAAAATTGCAG 3' and reverse, 5' TTTTGTAGGTCTCTTGGTATGTG 3'; Aurora B: forward, 5' ATGGCCCAGAAGGAGAACTCCTAC 3' and reverse, 5' GTAGAGACGCAGGATGTTGGGATG 3'; and α-actin: forward, 5' AGCGGGAAATCGTGCGTG 3' and reverse, 5' CAGGGTACATGGTGGTGC 3'. To amplify the 4 alternatively spliced forms of the C-terminal region of hNinein, we used primer combinations that included the hNinein forward primer, 5' CAGCTGCTTTGGCAAGAGAATGA 3', and reverse primers for isoform 1, 5' TCACAGGTGCCCAATCCTTCTG 3', isoform 2, 5' CTATGACCTCAAAGGAGGTGTAG 3', isoform 5, 5' TTAATGGCAATAAAGGGATGTAAA 3', and isoform 6, 5' CTACTTCCAACCACTGAGTT 3'.

The expression level was estimated by densitometer, +++ abundant; ++ moderate; + rare; - absent.

### Immunohistochemistry

Frozen tissue samples were cryosectioned, fixed with acetone at room temperature for 40 minutes, and incubated with monoclonal antibodies specific for Aurora A (GTU-88; Sigma, St. Louis, MO). Aurora A expression was then visualized using the alkaline phosphatase anti-alkaline phosphatase method [24].

### Confocal microscopy

Antibodies specific for γ-tubulin (Sigma), hNinein (pAb) (our preparation, rabbit, hNinein 1617_1931 a.a.), and Aurora A (pAb) (Sigma) were diluted in PBS containing 1% bovine serum albumin. Fluorescent secondary antibodies were diluted 1:200 in PBS with 1% BSA and 5% goat normal serum.

GBM8401 glioma cells [25] were purchased from American Type Culture Collection (ATCC, Manassas, VA) and cultured on glass coverslips in RPMI medium (Gibco) supplemented with 10% fetal bovine serum, 1% nonessential amino acids (Gibco), 100 IU/ml penicillin, and 100 μg/ml streptomycin (Gibco) at 37°C in a humidified 5% CO2 incubator for 24 hr. For synchronization, cells were treated with 200 ng/ml nocodazadole (Sigma). The coverslips were then washed twice with PBS, fixed with ice-cold methanol for 4 min at 4°C, and washed twice with PBS. For immunofluorescence of glioma tissues, frozen sections were prepared as above.

The coverslips with glioma cells or slides with histology sections were then incubated with primary antibody for 1 h at room temperature and washed twice in PBS for 5 min. After incubation at room temperature for 30 min with a fluorescent (Texas red or fluorescein isothiocyanate)-conjugated secondary antibody (Invitrogen) and DAPI (Roche) staining, the specimens were washed twice in PBS for 5 min. The coverslips were then dried and mounted onto glass slides, and the slides were observed by confocal microscopy (PM-20 camera; Olympus, Tokyo, Japan; MRC-1024 laser confocal system; Bio-Rad, Hercules, CA).

### Statistical analysis

Categorical variables, including four-level expressions and two-level expressions compared to normal brain tissue, were presented as counts and percentages. Fisher's exact test was performed to test the independence between the categorical variables and the two groups: high- and low-grade gliomas. A *p*-value < 0.05 was considered statistically significant. Statistical analyses were performed using SPSS 15.0 statistical software (SPSS Inc., Chicago, IL).

## Results

**Figure 1:**
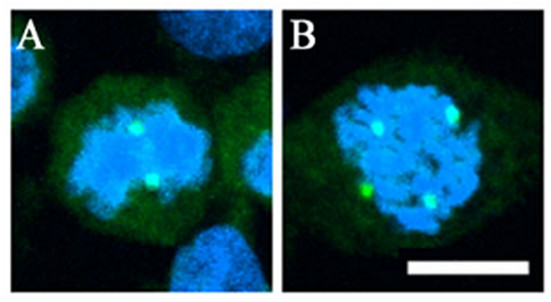
**Centrosome defects in the glioma cell line, GBM8401**. Centrosomes were visualized with antibodies specific for γ-tubulin (green). DNA was stained with DAPI (blue). Bar: 10 μm, magnification 2000 ×. A, Bipolar spindles. B, Multipolar spindles.

Using confocal microscopy to visualize γ-tubulin, a major structural protein of the centrosome, multiple centrosomes were observed in GBM8401 cells (Fig. 1). Notably, the centrosome number was not consistent from cell to cell, reflecting multiple genetic clones within the cell line and/or an inability of the glioma cells to regulate centrosome number. In synchronized GBM4801 glioma cells, multiple centrosomes were observed in 20% of the synchronized cells (Fig. 1B). When the GBM8401 cells were originally described, the cell line had a near-diploid 48, XX karyotype [25].

**Figure 2:**
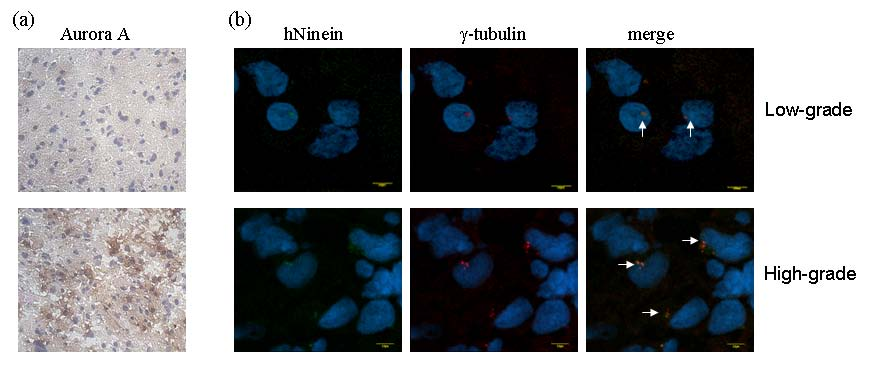
**Aurora A, hNinein, and γ-tubulin expression in low- versus high-grade glioma**. *A*, Immunohistochemical analysis of Aurora A expression (brown), 200× magnification. *B*, hNinein (green) and γ-tubulin (red), counterstained for DNA with DAPI (blue) was assessed by confocal microscopy. Bars = 10 μm, 1000× magnification.

Centrosomes were visualized in the glioma samples using confocal microscopy of tissue sections to visualize γ-tubulin and hNinein expression (Fig. 2B). In low-grade gliomas, the two centrioles of the centrosome were faintly visible and the nuclei were morphologically normal (Fig. 2B, upper panels). In contrast, high-grade gliomas had greater than two, often very brightly staining centrioles within large, atypical nuclei (Fig. 2B, lower panels). These enlarged nuclei with multiple centrioles were seen in the glioma cell line (Fig. 1B).

Immunohistochemical analysis of Aurora A in the glioma samples revealed little-or-no expression in low-grade gliomas (Fig. 2A, upper panel); however, moderate-to-strong Aurora A staining in the high-grade gliomas was observed (Fig. 2A, lower panel). The presence of high Aurora A expression levels corresponded with the nuclear and centrosomal abnormalities in the high-grade gliomas.

**Figure 3:**
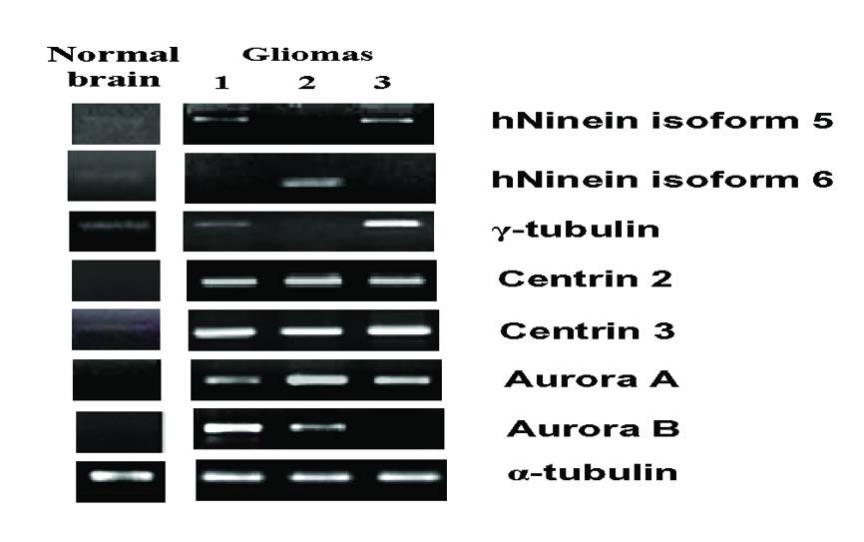
**RT-PCR analysis of hNinein isoforms 5 and 6, γ-tubulin, Aurora A, and Aurora B in gliomas**. α-tubulin expression was used as an internal loading control. Data from three gliomas are shown compared to a normal human brain sample. Data shown are representative of at least three independent experiments per glioma.

Having established that the level of Aurora A, a mitotic regulatory protein, and centrosome abnormalities, which are affected by Aurora A, were elevated in high- but not low-grade gliomas, we used RT-PCR to analyze the mRNA expression levels of a panel of regulatory and structural proteins of centrosomes (Fig. 3). In normal human brain tissue, the mRNA expression levels for centrosomal structural proteins, including centrin 3, γ-tubulin, and hNinein isoforms 1, 2, 5 and 6, was low; there was no detectable expression of Aurora A and Aurora B mRNA. In gliomas, most of the mRNAs analyzed in the panel were elevated to varying degrees (Fig. 3).

In order to identify mRNAs common to all high-grade gliomas, tissue from a total of 34 glioma patients was collected and divided into high-grade (n = 23) and low-grade glioma (n = 11) groups, according to the WHO guideline [23]. In addition to analyzing the glioma specimens, the expression profiles were also analyzed in normal brain tissue, and were characterized as absent (-), rare (+), moderate (++), and abundant (+++), according to the expression intensities. In normal brain tissue, expression of hNinein isoforms 1, 2, 5, and 6, γ-tubulin, and centrin 3 was rare whereas Aurora A, Aurora B, and centrin 2 mRNA expression was absent (data not shown). As shown in Table 1, significant different expression were observed between high- and low-grade glioma in both γ-tubulin and Aurora A mRNA s. Specifically, in low-grade glioma group, γ-tubulin expression was rare in 81.8% (9/11) and moderate in 18.2% (2/11) of the samples. However, in the high-grade glioma group, 60.9% (14/23) and 14.4% (4/23) displayed moderate and abundant γ-tubulin expression, respectively. In addition, in the low-grade glioma group, Aurora A expression was absent in 21.3% (3/11) of the samples and rare in 72.7% (8/11); however, in the high-grade group, no sample showed absent of Aurora A expression, and its expression was rare in 87% (20/23) and moderate in 13.0% (3/23) of the samples.

**Table 1:** Predictive value of relative mRNA expression levels for identifying high-grade gliomas

		Group				
			
mRNA		Low-grade glioma (*n *= 11)		High-grade glioma (*n *= 23)		*p*-value
hNinein isoform 1	Absent	0	(0%)	1	(4.3%)	0.741
	Rare	9	(81.8%)	13	(56.5%)	
	Moderate	2	(18.2%)	8	(34.8%)	
	Abundant	0	(0%)	1	(4.3%)	
hNinein isoform 2	Absent	2	(18.2%)	0	(0%)	0.254
	Rare	7	(63.6%)	18	(78.3%)	
	Moderate	2	(18.2%)	4	(17.4%)	
	Abundant	0	(0%)	1	(4.3%)	
hNinein isoform 5	Absent	1	(9.1%)	0	(0%)	0.501
	Rare	8	(72.7%)	15	(65.2%)	
	Moderate	2	(18.2%)	7	(30.4%)	
	Abundant	0	(0%)	1	(4.3%)	
hNinein isoform 6	Absent	3	(27.3%)	14	(60.9%)	0.141
	Rare	8	(72.7%)	9	(39.1%)	
	Moderate	0	(0%)	0	(0%)	
	Abundant	0	(0%)	0	(0%)	
γ-tubulin	Absent	0	(0%)	0	(0%)	0.005*
	Rare	9	(81.8%)	5	(21.7%)	
	Moderate	2	(18.2%)	14	(60.9%)	
	Abundant	0	(0%)	4	(17.4%)	
Aurora A	Absent	3	(27.3%)	0	(0%)	0.030*
	Rare	8	(72.7%)	20	(87.0%)	
	Moderate	0	(0%)	3	(13.0%)	
	Abundant	0	(0%)	0	(0%)	
Aurora B	Absent	3	(27.3%)	4	(17.4%)	0.653
	Rare	6	(54.5%)	16	(69.6%)	
	Moderate	2	(18.2%)	3	(13.0%)	
	Abundant	0	(0%)	0	(0%)	
Centrin 2	Absent	2	(18.2%)	1	(4.3%)	0.235
	Rare	2	(18.2%)	6	(26.1%)	
	Moderate	5	(45.5%)	15	(65.2%)	
	Abundant	2	(18.2%)	1	(4.3%)	
Centrin 3	Absent	1	(9.1%)	1	(4.3%)	0.536
	Rare	3	(27.3%)	3	(13.0%)	
	Moderate	3	(27.3%)	5	(21.7%)	
	Abundant	4	(36.4%)	14	(60.9%)	

NOTE: In normal brain tissue Aurora A, Aurora B, and Centrin 2 mRNAs were absent and hNinein isoforms 1, 2, 5, and 6, γ-tubulin, and Centrin 3 mRNAs were rare.

* *P *< 0.05 by Fisher's exact test

As shown in Table 2, the mRNA expression levels of structural and regulatory centrosomal proteins in low- and high-grade gliomas were compared relative to levels observed in normal brain tissue. The mRNA expression levels were divided into two groups, "normal" and "higher than normal", based upon its expression relative to that observed in normal human brain tissue. In the high-grade glioma group, 78.6% (18/23) of the samples had higher than normal expression of γ-tubulin mRNA, which was significantly higher than in the low-grade glioma group (18.2%, 2/11, p < 0.05).

**Table 2:** Predictive value of abnormal mRNA levels for identifying high-grade glioma

		Group				
			
mRNA		Low-grade glioma (*n *= 11)		High-grade glioma (*n *= 23)		*p*-value
hNinein isoform 1	>Normal	2	(18.2%)	9	(39.1%)	0.274
	Normal	9	(81.8%)	14	(60.9%)	
hNinein isoform 2	>Normal	2	(18.2%)	5	(21.7%)	1.000
	Normal	9	(81.8%)	18	(78.3%)	
hNinein isoform 5	>Normal	2	(18.2%)	8	(34.8%)	0.438
	Normal	9	(81.8%)	15	(65.2%)	
hNinein isoform 6	<Normal	3	(27.3%)	14	(60.9%)	*
	Normal	8	(72.7%)	9	(39.1%)	
γ-tubulin	>Normal	2	(18.2%)	18	(78.3%)	0.002^†^
	Normal	9	(81.8%)	5	(21.7%)	
Aurora A	>Normal	0	(0%)	3	(13.0%)	0.535
	Normal	11	(100.0%)	20	(87.0%)	
Aurora B	>Normal	2	(18.2%)	3	(13.0%)	1.000
	Normal	9	(81.8%)	20	(87.0%)	
Centrin 2	>Normal	7	(63.6%)	16	(69.6%)	1.000
	Normal	4	(36.4%)	7	(30.4%)	
Centrin 3	>Normal	7	(63.6%)	19	(82.6%)	0.388
	Normal	4	(36.4%)	4	(17.4%)	

NOTE: "Normal" is equivalent to the level of mRNA expression observed in the normal brain tissue.

* No *p*-value could be calculated because neither group showed a moderate or abundant increase in mRNA expression (see Table 1).

^† ^*P *< 0.05 by Fisher's exact test

## Discussion

To determine if centrosome-related protein expression was altered in gliomas and if it corresponded with gliomal staging, 34 glioma patient samples as well as GBM8401 cells were analyzed. Supernumerary centrosomes and abnormal nuclei were detected in GBM8401 cells and in high- but not low-grade gliomas. Elevated Aurora A expression was also observed specifically in high-grade gliomas. In addition, increased hNinein isoform5, hNinein isoform 6, γ-tubulin, Aurora A, and Aurora B mRNA expression was observed in gliomas as compared to normal brain. Finally, γ-tubulin and Aurora A mRNA levels significantly increased with glioma grade.

Over 100 years ago, researchers proposed the idea that supernumerary centrosomes could lead to abnormal chromosome segregation and cancer [26]. In 1982, Friedlander used electron microscopy to explore this possibility in gliomas; however, few supernumerary centrioles were observed, although clusters of centrioles were occasionally observed [27]. Using antibodies specific to centrosome proteins, we frequently observed supernumerary centrosomes in the giant nuclei of high-grade glioma samples and in GBM8401 cells, which is in agreement with similar studies in other cancer types [2,28].

RT-PCR analysis of centrosomal protein expression revealed varying degrees of upregulated expression as compared to normal brain tissue. Each glioma may have acquired different types of genetic (or epigenetic) changes that were all related to the same selective pressure to alter mitotic regulation.

Although different RNA profiles were observed for each glioma analyzed, our theory--that there is a single underlying trait that is being selectively altered-- suggests that the expression of certain key proteins would be altered in all high-grade gliomas because their upregulation is a necessary consequence of removing the mitotic checkpoints. Therefore, we analyzed the raw data to identify RNAs that were significantly elevated in all the glioma samples, presuming these to code for key proteins, and thus be most reliable as markers for future development of diagnostic tests or therapeutic interventions. Of the 34 gliomas, we found that elevated Aurora A and γ-tubulin mRNAs were significantly associated with all high-grade gliomas all of which displayed mitotic dysregulation. Thus, regardless of the underlying mechanism by which each glioma acquired altered mitotic regulation, they all had elevated Aurora A and γ-tubulin. In the more stringent two-level statistical test, Aurora A expression alone was a significant predictor of the glioma grade. Considering that γ-tubulin levels are likely a downstream effect of Aurora A dysregulation, the lack of a significant result in the two-level test does not make changes in γ-tubulin completely irrelevant.

Aurora A, which is also known as serine/threonine kinase 15 (STK15), is a key regulatory protein controlling centrosome maturation, spindle assembly, and chromosome segregation [29]. An Aurora A gene mutation is characterized by a centrosome separation defect [30]. In addition, mutations in the Increase for Ploidy 1(Ipl1) gene in *Saccharomyces cerevisiae*, that is closely homologous to Aurora A, resulted in chromosome segregation defects [31]. Furthermore, overexpression of Aurora A induced centrosome amplification, aneuploidy, and transformation [32]. Human Aurora-related kinases were indeed overexpressed in several human cancer types [33]. Although the effects of Aurora A overexpression and gene mutation have been well-characterized in many cancers, its influence on cancer progression and therefore grade are not as clear. Klein *et al*. [12] performed a similar study of Aurora A mRNA expression in a panel of low- and high-grade glioma samples and showed that, however, STK15/Aurora A was overexpressed in 60% of the tumors analyzed, it was not predictive of glioma grade, which is inconsistent with our data.

Although the sample size of this study is a necessary limitation due to the difficulty in obtaining these samples, analysis of human tissue rather than a cell line is more clinically meaningful. In addition, this study supports future exploration of these potential markers in more extensive samplings as well as in other model systems. For example, GBM8401 cells may provide a useful tool to study the effects of altered Aurora A and γ-tubulin levels on supernumerary centrosomes, aneuploidy, or cellular transformation.

## Conclusions

γ-tubulin and Aurora A mRNA levels were significantly higher in high- versus low-grade gliomas. Markers for mitotic dysregulation, such as supernumerary centrosomes and altered centrosome-related mRNA and protein expression, were observed in gliomas as compared to normal tissue. Elevated mitotic regulatory proteins, such as Aurora A, and altered levels of centrosome structural proteins are common in many cancers, and treatments may soon be developed which act on these classes of proteins [3,4,29]. These results are clinically useful for glioma staging as well as the development of novel treatment strategies.

## Abbreviations

CHFR: checkpoint with forkhead and ring finger domails; Plk: Polo-like kinases; NIMA: Nek kinases; ATCC: American Type Culture Collection; Ipl1: Increase for Ploidy 1

## Competing interests

The authors declare that they have no competing interests.

## Authors' contributions

JKL: carried out the molecular genetic studies, participated in the sequence alignment, draft the manuscript. ASL: carrid out the molecular genetic studies and the immunoassay. CHC: carrid out the molecular genetic studies. FYL: carried out the molecular genetic studies. CHW: carried out the molecular genetic studies. SLH: participated in the design of the study. CCC: participated in the design of the study. YRH: conceived of the study, and participated in its design and helped to draft the manuscript. All authors read and approved the final manuscript

## Pre-publication history

The pre-publication history for this paper can be accessed here:

http://www.biomedcentral.com/1471-2407/10/268/prepub
